# Ivermectin and gynecologic cancer: What’s the data?

**DOI:** 10.1016/j.gore.2025.101803

**Published:** 2025-07-11

**Authors:** Vaidehi Mujumdar, Marilyn Huang, Lindsey Chippendale Smith, Fernanda Musa

**Affiliations:** a Atrium Health Levine Cancer Institute, 1021 Morehead Drive, Suite 2100, Charlotte, NC 28202, USA; b University of Virginia School of Medicine, P.O. Box 800712, Charlottesville, VA 22908, USA; c University of Virginia Health, 1215 Lee Street, Charlottesville, VA 22903, USA; d Providence-Swedish Cancer Institute, 1101 Madison St Suite 1500, Seattle, WA 98104, USA

**Keywords:** Ivermectin, COVID-19, Gynecologic cancers, Wonder drug, Anti-helminthic

## Abstract

• Ivermectin is currently approved by the US Food and Drug Administration (FDA) in 1996 as an oral medication for intestinal strongyloidiasis and onchocerciasis.
• The data on ivermectin as a gynecologic cancer-fighting compound is lacking.
• Clinical studies on ivermectin use in cancer are limited to effects observed in cell lines.
• We have not assessed ivermectin’s safety and efficacy in gynecologic cancers.
• We do not recommend and strongly caution the use of ivermectin in the treatment of gynecologic cancers.

## 1 Introduction

While medical misinformation and distrust in healthcare providers are certainly not a new phenomenon, accessibility to information has reached an unprecedented scale, outpacing the development of tools to help users evaluate the quality of the data they are consuming. During the COVID-19 pandemic, ivermectin was popularized not only for COVID-19 but also as a potential cancer therapy (Food and Administration, 2024). We have each been personally impacted by the loss of patients who chose ivermectin therapy over our best evidence-based advice and felt that a critical assessment of the existing evidence as it pertains to cancer was an unmet need.

Ivermectin is currently approved by the US Food and Drug Administration (FDA) in 1996 as an oral medication for intestinal strongyloidiasis and onchocerciasis (Food and Administration, 2024). As an anthelmintic medication, it has been successfully used to treat veterinary infections and other human parasitic infections such as lice and scabies as topical formulations. Increasingly, ivermectin is being heralded as a wonder drug across social media platforms from curing Stage IV uterine cancer to treating cancer-causing parasites and a whole host of malignancies including brain and thyroid cancers (Lawrence, 2025, Blum and Can, 2025) We find ourselves answering questions about the utility of ivermectin being used either with chemotherapy or as an alternative to chemotherapy. Patients frequently bring multiple scientific and non-scientific articles to clinic asking for evaluation and recommendations. If we are fortunate enough to have clinical pharmacists, they are frequently asked to review page-long supplement lists to ensure safety while on treatment. We question, what is the evidence behind the hype of this so-called wonder drug? In this manuscript we focus on the existing evidence regarding ivermectin in the treatment of cancer, specifically those arising from the gynecologic tract.

## 2 History of ivermectin and recent data

Ivermectin was discovered in the 1970s in Tokyo, Japan by microbiologist Satoshi Omura and Irish parasitologist William C. Campbell (Dy and Juangco, 2023). The pair studied soil samples for active medicinal compounds in partnership with a pharmaceutical company. They came across a soil sample containing *Streptomyces avermictilis*, which is effective against parasitic worms. The drug was purified into a safe and stable compound that was used in the treatment of river blindness and lymphatic filariasis in the mid-1980s and rendered Omura and colleagues the Nobel Prize in 2015 (Crump and Ōmura, 2011).

Re-purposing already approved drugs facilitates more rapid access to treatment by building on existing safety and efficacy data. During the early phase of the COVID-19 pandemic ivermectin re-surged as a potential treatment for the viral illness (Santin et al., 2021). Ivermectin has been proposed to have direct action against SARS-CoV-2, antiviral properties, modulating inflammatory targets (interferon levels, toll-like receptors, etc), and purported to target host plasmin, annexin A2, and CD 147 (Santin et al., 2021). By 2021, 20 randomized clinical trials and seven *meta*-analyses had been published suggesting the benefit of ivermectin in the treatment of COVID-19. Using insurance claims data by August 2021, ivermectin prescriptions peaked at more than 10 times higher than pre-pandemic rates leading to a shortage of the drug (Zaidi and Dehgani-Mobaraki, 2022) Since then, several studies have debunked the use of ivermectin for COVID-19 including a multi-institute United States based randomized clinical trial (RCT) (Rockwell et al., 2025, Reis et al., 2022, Hayward et al., 2024, Naggie et al., 2023). One RCT showed that treatment with ivermectin did not result in lower incidence of hospitalization due to COVID-19 progression nor did it lower incidence of prolonged emergency department observation for outpatients with COVID-19 (Reis et al., 2022) Similarly, the PRINCIPLE trial concluded that ivermectin was unlikely to provide any clinical benefit in the recovery, hospital admissions or long-term outcomes of patients with COVID-19 (Hayward et al., 2024). The ACTIV-6 trial studied the use of ivermectin in outpatients with mild to moderate COVID-19 and concluded that ivermectin did not improve time to sustained recovery (Naggie et al., 2023).

The data on ivermectin as a gynecologic anti-cancer therapy is lacking. Jawad and Richardson (2023) and Nunes and Ricardo (2022) studied the role of ivermectin to synergistically work with pitavastatin and paclitaxel, respectively in chemo-resistant ovarian cancer cell lines (Jawad and Richardson, 2023, Nunes and Ricardo, 2024). Jawad and Richardson (2023) tested combinations of pitavastatin with ivermectin in six ovarian cancer cell lines. When the drugs were combined and assessed in cell growth assays, ivermectin potentiated the reduction of cell viability in one cell line up to 25 % (Jawad and Richardson, 2023). Nunes and Ricardo (2023) demonstrated that paclitaxel with pitavastatin or ivermectin showed the highest cytotoxic effect by IC_50_ and the strongest synergism for two high grade serous ovarian cancer chemoresistant cell lines (Nunes and Ricardo, 2024). This resulted in a chemotherapeutic effect superior to both drugs alone (Nunes and Ricardo, 2024). The mechanism of action is purported to be via mTOR pathway inhibition (Jawad and Richardson, 2023, Nunes and Ricardo, 2024, Zhang et al., 2020) which is intriguing, as this is a pathway that has been targeted in both endometrial cancers and perivascular epithelioid cell tumors (PEComas) (Slomovitz et al., 2022, Wagner et al., 2021) Ivermectin has also been shown to block PAK1, an oncogenic kinase, in human ovarian cancer and NF2-deficient Schwannoma cell lines to suppress PAK1-dependent growth in cell culture (Hashimoto et al., 2009).

The existing literature focuses mostly on in-vitro cell models and organoids not related to gynecologic malignancies (Jiang et al., 2019, Lee et al., 2022). Lu Jiang et al. 2019 showed that ivermectin reversed the resistance to vincristine and adriamycin in two solid tumor cell lines (colorectal and breast) and one hematologic tumor cell line chronic myeloid leukemia) both in vitro and in vivo (Jiang et al., 2019). By mechanism, ivermectin reduced the expression of P-glycoprotein (P-gp) via inhibiting the epidermal growth factor receptor (EGFR) (Jiang et al., 2019). Lee et al. 2022 tested ivermectin on pancreatic cancer proliferation using patient-derived organoids. Ivermectin significantly inhibited the growth of organoids in a concentration-dependent manner suggesting that ivermectin inhibits the growth of pancreatic cancer (Lee et al., 2022). Another preclinical study demonstrated ivermectin and sorafenib were synergistic in hepatocellular carcinoma in vitro and in vivo models (Lu et al., 2022). However, findings in non-human models often do not translate into human efficacy due to the complexity of human physiology. Another prior study has suggested that ivermectin induces cytostatic autophagy by blocking the PAK1/AKT axis in breast cancer (Wang et al., 2016). There is currently only one clinical trial listed on ClinicalTrials.gov that investigates the use of ivermectin in humans. This is a phase II study looking at the combination of ivermectin in combination with balstilimab or pembrolizumab in patients with metastatic triple negative breast cancer (NCT05318469) (xxxx, 2022) There are no other trials evaluating ivermectin as a cancer treatment listed on the National Cancer Institute’s website (Find NCI-Supported Clinical Trials, 2025).

## 3 FDA approved dosing information

Since FDA approval, ivermectin was mostly used as an anti-parasitic for animals and humans. Ivermectin is also formulated in some prescription-only topical lice treatments (Mayo Clinic, 2025) For river blindness, the dosing is weight-based in adults and children. For patients weighing 15 kg (kg) or more, the dose is usually 150 Âµg (mcg) per kg taken as a single dose tablet. Each tablet contains 3 mg (mg) of ivermectin. For threadworms, the dosing for anyone weighing over 15 kg more is typically 200 mcg per kg (Mayo Clinic, 2025, U.S. Food and Drug Administration, Center for Drug Evaluation and Research. ANDA NDA 204154, 2025) For head lice and skin infections, the FDA notes that each gram of topical ivermectin at 0.5 % of lotion contains 5 mg of ivermectin. The instructions are to apply lotion to dry hair or skin in a sufficient amount (Mayo Clinic, 2025, U.S. Food and Drug Administration, Center for Drug Evaluation and Research. ANDA NDA 204154, 2025).

## 4 Toxicity

### 4.1 The FDA approved the use of ivermectin

The approved dosing for ivermectin for parasitic infections is somewhere between 150–200 mcg/kg in the tablet form for individuals who weigh greater than 15 kg (Mayo Clinic, 2025, U.S. Food and Drug Administration, Center for Drug Evaluation and Research. ANDA NDA 204154, 2025). A mini-review by Johson-Arbor in Clinical Toxicology in 2022 suggests that the effects of ivermectin after therapeutic oral use include edema, rash, headache, and ocular disturbances. Ivermectin can also interact with blood thinners (U.S. Food and Drug Administration, Center for Drug Evaluation and Research. Sklice NDA, 2025). A case report describes a patient who developed large hematoma under his tongue while taking warfarin and ivermectin together (Johnson-Arbor, 2022). Another case report suggests multifactorial encephalopathy in a 52-year-old male who was using supratherapeutic doses of ivermectin in the Philippines (Gilbert and Slechta, 2018). Another case report of a young woman in Cameroon described a neurological disorder after intoxication with about 400 tablets of ivermectin 3 mg, which is 100 times the standard doses used for parasitic infections (Donfo-Azafack et al., 2023). The FDA approved doses are typically one-time or two doses total. Extrapolating from approved doses to oncologic settings at higher and/or more frequent doses is not evidence-based and experimental at best. The use of ivermectin in gynecologic oncology should only be done in a clinical trial setting with safety measures (Office for Human Research Protections (OHRP), 2025). It is also challenging to speak of interactions between drugs that have not been studied. On one hand, ivermectin could promote faster clearance of chemotherapy or other cancer-fighting compounds, lowering their toxicity; but the counter is that it could also slow it down, potentiating toxicity and the emergence of new and possibly severe side effects seen in several case reports around the world (Rendic, 2021).

## 5 Conclusions

Investigations on the use of ivermectin in cancer are limited to pre-clinical studies utilizing cell culture and anima models. To date, there are no clinical trials of human subjects with gynecologic cancers; thus, it is not possible to assess the safety and efficacy of ivermectin or to make educated hypotheses on its superiority to the current standard of care. Although various alternative strategies for cancer treatment exist, the lack of rigorous evidence precludes any ethical recommendations of ivermectin in the treatment of gynecologic malignancies. The safety and efficacy of ivermectin in gyn cancers is unknown and unsupported. We recommend against the use of ivermectin in the treatment of gynecologic cancers.

## CRediT authorship contribution statement

**Vaidehi Mujumdar:** Writing – review & editing, Writing – original draft, Validation, Investigation, Formal analysis, Data curation, Conceptualization. **Marilyn Huang:** Writing – review & editing, Writing – original draft, Validation, Supervision, Data curation, Conceptualization. **Lindsey Chippendale Smith:** Writing – review & editing, Investigation, Data curation, Conceptualization. **Fernanda Musa:** Writing – review & editing, Writing – original draft, Validation, Supervision, Investigation, Data curation, Conceptualization.

## Declaration of Competing Interest

The authors declare that they have no known competing financial interests or personal relationships that could have appeared to influence the work reported in this paper.
